# Relationship Between Epidermal Matrix Metalloproteinase-1 and Dermal Collagen Reduction in Skin Subjected to Chronic Sun Exposure

**DOI:** 10.3390/jcm14051433

**Published:** 2025-02-20

**Authors:** Ushio Hanai, Keigo Kawabata, Yotaro Tsunoda, Hitoshi Nemoto, Kotaro Imagawa, Ayumi Kusaka-Kikushima, Yoshito Takahashi, Hiroyuki Yoshida, Tadashi Akamatsu

**Affiliations:** 1 Department of Plastic Surgery, Tokai University School of Medicine, Isehara 259-1143, Japan; tsunohachi@gmail.com (Y.T.); hitonemo7plas@gmail.com (H.N.); imagawa@tokai.ac.jp (K.I.); at7071@tokai.ac.jp (T.A.); 2 Biological Science Research, Kao Corporation, Odawara 250-0002, Japan; kawabata.keigo@kao.com (K.K.); kusaka.ayumi@kao.com (A.K.-K.); takahashi.yoshito@kao.com (Y.T.); yoshida.hiroyuki2@kao.com (H.Y.)

**Keywords:** actinic elastosis, chronic sunlight exposure, matrix metalloproteinase-1, photoaging, tissue remodeling

## Abstract

**Background/Objectives:** Temporary decreases in dermal collagen caused by artificial ultraviolet exposure are largely affected by increased epidermis-derived matrix metalloproteinase (MMP)-1 levels. However, the role of epidermal MMP-1 in dermal tissue remodeling induced by chronic sun exposure remains unclear. This study aimed to clarify the involvement of epidermal and dermal MMP-1 in dermal collagen reduction induced by chronic sun exposure. **Methods:** Immunofluorescent staining of 30 facial skin tissue samples was performed to visualize MMP-1. The fluorescence intensity of epidermal MMP-1 observed on microscopic images was analyzed in relation to the severity of dermal tissue remodeling and the dermal collagen fiber density. A similar correlation analysis of the number of dermal MMP-1-positive cells was also performed. **Results:** Epidermal MMP-1 was observed in the stratum spinosum of skin without severe tissue remodeling; however, in skin with severe dermal tissue remodeling, MMP-1 was localized throughout the epidermis. The epidermal MMP-1 signal area and dermal collagen fiber density were negatively correlated (*ρ* = −0.383; p = 0.0002; n = 90). However, the ratio of dermal MMP-1-positive cells to total dermal cells was only negatively correlated with the collagen fiber density in skin that was not severely remodeled (*ρ* = −0.746; p = 0.001; n = 15). **Conclusions:** Epidermal MMP-1 is involved in the tissue remodeling of skin that is subjected to chronic sun exposure and short-term ultraviolet radiation exposure. However, dermal-cell-derived MMP-1 may be involved in biological processes that require an immediate collagen degradation response. The results of this study demonstrate the importance of controlling epidermal MMP-1 to inhibit dermal tissue remodeling induced by chronic sun exposure and provide new insights that are beneficial to the development of anti-photoaging skincare cosmetics.

## 1. Introduction

Excessive exposure to solar ultraviolet (UV) radiation has detrimental effects on the function and appearance of skin. Acute exposure can induce skin inflammation, such as erythema and sunburn, and chronic exposure can accelerate the signs of aging, such as wrinkles and sagging. Skin aging induced by solar UV radiation is referred to as photoaging. One of the major characteristics of photoaged tissue caused by chronic sun exposure is remodeled dermal collagen and elastic fiber structures (actinic elastosis) [1,2]. Tissue remodeling is considered a key factor in the development of wrinkles and sagging, because it results in skin property alterations [3,4]. Past reports have classified the severity of tissue remodeling into six photoaging stages (stages I–VI), and a significant positive correlation between these stages and the appearance of wrinkles has been identified [4]. Therefore, understanding the mechanisms underlying tissue remodeling is considered highly important from a cosmetic perspective.

Dermal tissue remodeling during photoaging involves the reduction in collagen fibers by matrix metalloproteinases (MMPs) [5]. Excessive MMPs have been shown to disrupt the structural integrity of the epidermis and dermis, leading to photoaging [6,7]. When dermal collagen is cleaved by MMP-1 (collagenase), it is further degraded by MMP-3 (stromelysin) and MMP-9 (gelatinase) [8]. Therefore, the upregulation of MMP-1 production plays a significant role in the reduction in collagen fibers during UV-induced tissue remodeling.

Studies by Fisher et al. [5] and Quan et al. [9] have shown that epidermal keratinocytes are the primary cells responsible for promoting MMP-1 production, and that MMP-1 acts on collagen degradation after passing through the basement membrane to reach the dermis. Thus, it is speculated that MMP-1 that is released from epidermal keratinocytes is largely involved in dermal tissue remodeling induced by chronic sun exposure. However, the relationship between the progression of photoaging induced by daily UV exposure for more than a decade and the production of MMP-1 in the epidermis remains unclear because of the difficulty associated with obtaining a large number of skin tissue samples with different degrees of photoaging severity. In this study, 30 skin tissue samples, classified as photoaging stages I to VI according to the criteria reported by Kawabata et al. [4], were analyzed to investigate the relationship between changes in dermal collagen fiber density, the severity of photoaging, and the presence of MMP-1-positive cells in the epidermis. Additionally, the relationship between the ratio of dermal MMP-1-positive cells to the total number of dermal cells and the change in dermal collagenous fiber density was analyzed.

## 2. Materials and Methods

### 2.1. Skin Tissue Samples

Written informed consent was obtained from all study participants, and all experiments were approved on 29 August 2023 by the Tokai University Hospital Ethics Committee (23R074-001 MH). Twenty Japanese women (age range: 35–96 years) volunteered to participate in this study. The surgical margins of the biopsy specimens were obtained from the preauricular and/or postauricular skin of the participants. Some skin tissues were collected from the same regions of the same participant. A total of 30 skin tissue samples (5 samples classified as each photoaging stage) were obtained (Table 1).

### 2.2. Elastica van Gieson Stain and Photoaging Severity

**Figure 1:**
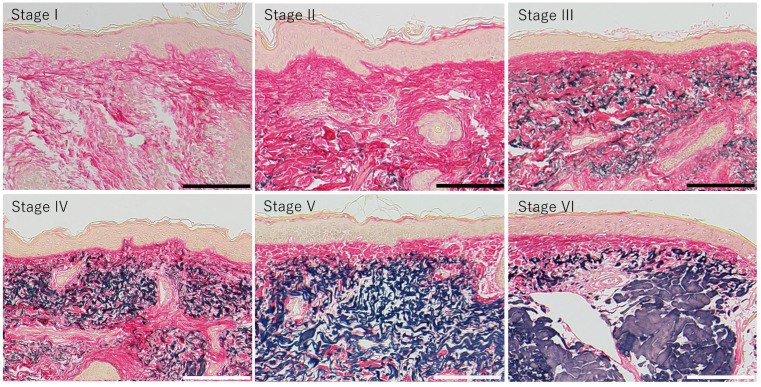
Skin tissues analyzed during this study. Skin tissues were stained with Elastica van Gieson to exhibit photoaging and dermal tissue remodeling and divided into photoaging stages I to VI according to the criteria reported by Kawabata et al. [4]. Collagen fibers and elastic fibers (or elastotic materials) are stained pink and black, respectively. Bars: 100 μm.

Biopsy specimens were fixed with Mildform 10N (Wako Pure Chemical Industries, Osaka, Japan) and embedded in paraffin blocks using routine techniques. Serial vertical sections of skin were sliced (thickness: 2 μm) with a microtome and mounted onto MAS-GP-coated slides (Matsunami Glass Industries, Osaka, Japan). Elastica van Gieson (EVG) staining was performed as described previously [4]. Images of EVG-stained tissues were scored according to the criteria reported by Kawabata et al. [4] and classified as one of six photoaging stages (Figure 1).

### 2.3. Masson’s Trichrome Staining

Serially sliced skin tissue sections were stained with Masson’s trichrome (MT). Following rehydration, the sections were incubated with a mixture of equal amounts of 10% potassium dichromate and 10% trichloroacetic acid for 10 min. After the sections were rinsed with running tap water, they were incubated in Wegert’s iron hematoxylin (40342 and 40352; Muto Pure Chemicals Co., Ltd., Tokyo, Japan) for 10 min to differentiate the nuclei; thereafter, they were incubated with 1% acetic acid. The sections were submerged in MT staining solution B (Ponceau xylidine acid fuchsin azophloxin solution; 40252; Muto Pure Chemicals Co., Ltd. Tokyo, Japan) for 20 min and then in 1% acetic acid to stain the cytoplasm and erythrocytes. Next, slides were treated with 2.5% phosphor-tungstic acid solution for 12 min as a mordant; immediately thereafter, they were submerged in three-times-distilled aniline blue and orange G solution (40051; Muto Pure Chemicals Co., Ltd.) for 10 min to stain the collagen fibers. The sections were subsequently washed with running water for 2 min and treated with 1% acetic acid solution for 1 min. Thereafter, the sections were dehydrated with ethanol, cleared with xylene, and mounted with Marinol (10781; Muto Pure Chemicals Co., Ltd., Tokyo, Japan).

### 2.4. Immunofluorescence Study

The remaining sections were labeled with MMP-1. After rehydration, sections were rinsed with Tris-buffered saline (TBS). The sections were autoclaved at 121 °C in 10 mmol/L citrate buffer (pH 6.0) for 10 min. Endogenous peroxidase activity was inactivated by incubation in methanol containing 0.3% hydrogen peroxidase (H_2_O_2_) for 30 min at room temperature. To block nonspecific binding, sections were incubated in 4% Block Ace (UK-B40; KAC Co., Ltd., Kyoto, Japan) in TBS-T (0.05% Tween 20 in TBS) for 60 min at room temperature. Then, polyclonal rabbit anti-MMP-1 (ab38929; Abcam, Cambridge, UK) at a dilution of 1:400 in 4% Block Ace in TBS-T was applied overnight at 4 °C as the primary antibody. After washing with TBS, sections were incubated with Rhodamine Red-X-conjugated donkey anti-rabbit IgG (711-295-152; Jackson Immuno Research, West Grove, PA, USA) at a dilution of 1:400 in 4% Block Ace in TBS-T for 1 h at room temperature, followed by additional rinsing with TBS. Sections were mounted using a mounting medium containing DAPI (ab104139; Abcam, Cambridge, UK). Isotype control antibodies at the same concentration as that of the primary antibody (normal rabbit IgG; AB-105-C; R&D Systems, Minneapolis, MN, USA) were used. Fluorescence signals were examined using a fluorescence microscope (Olympus BX53; Tokyo, Japan) equipped with a DP74-CU camera (Olympus, Tokyo, Japan).

### 2.5. Image Analysis

ImageJ software (version 1.54; National Institutes of Health, Bethesda, MD, USA) was used to determine the mean signal intensity of epidermal MMP-1 in each epidermal area of a 300 μm × 400 μm image. The number of dermal MMP-1-positive cells in the total number of DAPI-stained dermal cells, excluding the hair follicle cells, was determined using the same images.

The percentage of collagen fibers in the skin tissue sections was measured following MT staining. The plug-in Color Deconvolution2 (version 2.1) in ImageJ (version 1.54j) software was used to evaluate the areas of the images that were stained blue (collagen) [10]. The mean collagen occupancy rate was calculated from three 100 μm × 100 μm fields per sample. The 100 μm × 100 μm square areas were randomly selected by the evaluators, and the hair follicles, blood vessels, and grenz zones were excluded.

### 2.6. Statistical Analysis

A comparative analysis was conducted using SPSS statistical software (version 29.0 for Windows; IBM Corp., Armonk, NY, USA). Histological findings were compared using Tukey’s test (p < 0.05 was considered statistically significant). Correlations between histological findings were evaluated using Spearman’s correlation analysis (p < 0.05 was considered statistically significant).

## 3. Results

As the photoaging stage progressed, the epidermal MMP-1 signals became more widely distributed. We investigated the relationship between changes in MMP-1 localization in the epidermis and the severity of photoaging to determine the effect of epidermal MMP-1 on dermal tissue remodeling (Figure 1).

**Figure 2:**
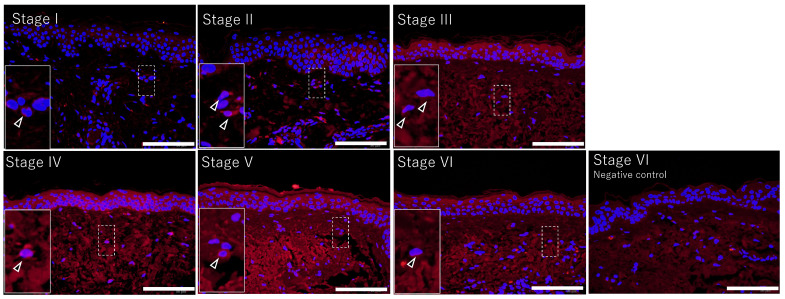
Localization of MMP-1 in the epidermis varies with the progression of photoaging-related dermal tissue remodeling. Typical image of MMP-1 at each photoaging stage I to VI. These are same area that was depicted in Figure 1. MMP-1 is visualized by immunofluorescence using an anti-MMP-1 antibody (red). Normal rabbit IgG was used as a negative control. Cell nuclei are counterstained with DAPI (blue). Bars: 100 μm. Insets: Highly magnified images of boxed areas. Arrowheads: Dermal MMP-1 positive cells.

Signals derived from anti-MMP-1 antibodies were detected in the epidermis by using immunofluorescence with anti-MMP-1 antibodies (Figure 2). Photoaging stage I exhibited a lower degree of dermal tissue remodeling, and weak signals derived from anti-MMP-1 were detected in the epidermal spinous layer. In photoaging stage II, signals were detected in the stratum granulosum and stratum spinosum. Some sites with very high signal intensity were also observed. However, such sites were rarely observed. Photoaging stages III and VI exhibited anti-MMP-1 signals that were detected throughout the epidermis (Figure 2). These findings suggest that epidermal MMP-1 is constitutively expressed in spinous cells and may extend to granular cells and basal cells as the photoaging stage progresses. Anti-MMP-1 signals were also detected in dermal cells and the extracellular matrix in skin classified as photoaging stages III and VI, but their expression and extracellular matrix types were not identified (Figure 2). These signals within the extracellular matrix were considered residues of MMP-1 that had been secreted by epidermal and/or dermal cells. No signal was detected in the epidermis when the isotype control antibody was used, and a weak positive signal was observed in the extracellular matrix of the dermis (Figure 2).

The area of epidermal MMP-1-positive cell expression was significantly increased in skin at more advanced stages of photoaging, and it was negatively correlated with the occupancy of dermal collagenous fibers.

**Figure 3:**
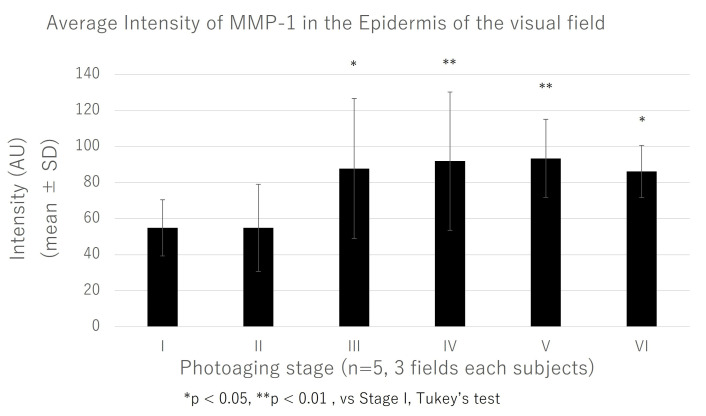
Comparison of epidermal MMP-1 signal intensity among six photoaging stages (stage I to VI). The mean signal intensity of epidermal MMP-1. Each epidermal area of the visual field was evaluated using ImageJ software at each photoaging stage. Graph data represent the mean ± SD of three fields of five subjects. AU, arbitrary unit; MMP-1, matrix metalloproteinase-1; SD, standard deviation.

The rate of epidermal MMP-1 signal intensity in each epidermal area was quantified through image analysis using ImageJ. In stages III, IV, V, and VI, the intensity of the epidermal MMP-1 signals was significantly higher than that in stage I (Figure 3).

**Figure 4:**
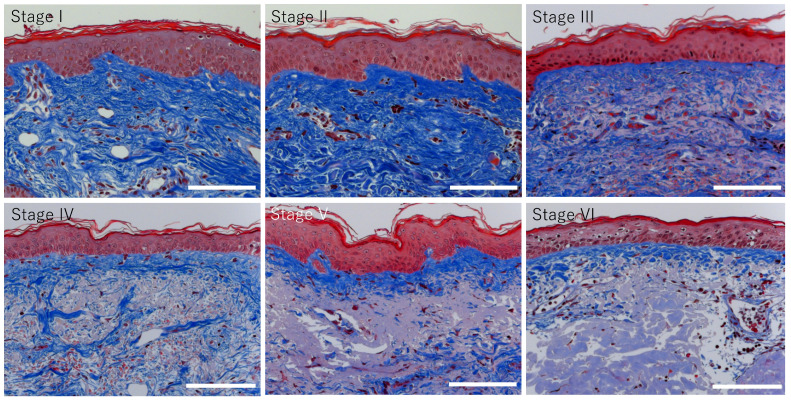
Changes in distribution of collagen fibers during varies with the progression of photoaging-related dermal tissue remodeling. Typical images of MMP-1 at each photoaging stage I to VI. Collagen fibers are stained by blue color with Masson’s trichrome (MT). Bars: 100 μm.

**Figure 5:**
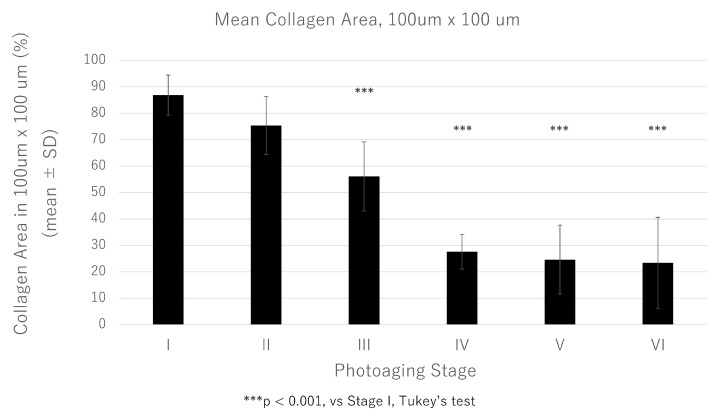
Comparison of collagen fiber areas in the dermis among six photoaging stages (stage I to VI). The presence of collagen fibers in randomly selected regions (100 μm × 100 μm) of the upper dermis, excluding the grenz zone, was evaluated using ImageJ software at each photoaging stage. Graph data represent the mean ± SD of the 100 μm × 100 μm fields of five subjects. SD, standard deviation.

Subsequently, the relationship between the epidermal MMP-1 signal intensity and quantitative changes in collagen fibers in the upper dermis was analyzed. Images of tissue that were stained with Masson’s trichrome (MT) revealed collagen fibers (stained blue), demonstrating histological features similar to those observed in photoaging stages using EVG staining. In other words, a significant reduction in the percentage of collagen fibers in randomly selected regions (100 μm × 100 μm) of the upper dermis, excluding the grenz zone, was observed at photoaging stages III to VI (Figure 4 and Figure 5). 

**Figure 6:**
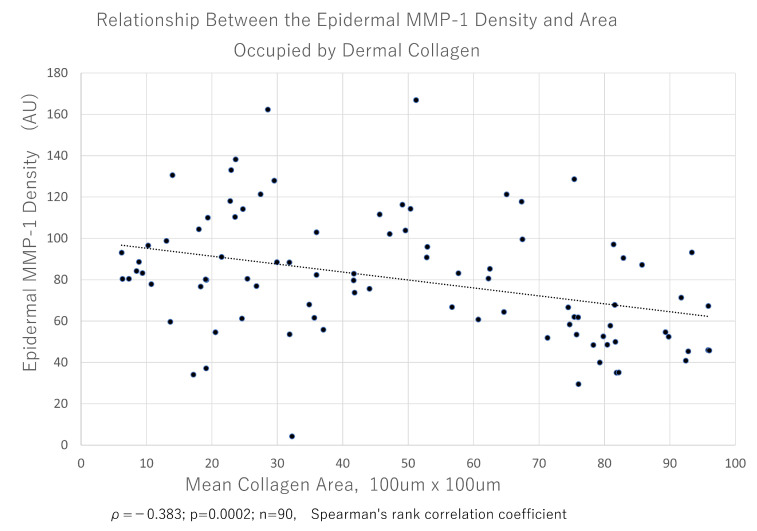
The relationship between the epidermal MMP-1 amount and dermal collagen fiber density. A scatter plot showing the relationship between the average MMP-1 signal intensity in the epidermis and mean dermal collagen occupancy rate of each sample. Dotted line represents the regression line. A significant negative correlation was detected between the two values based on the Spearman’s rank correlation coefficient. AU, arbitrary unit; MMP-1, matrix metalloproteinase-1.

The features of photoaging stages were reflected by these values; therefore, we analyzed the relationship between the epidermal MMP-1 signal intensity rate that we detected by immunofluorescence using anti-MMP-1 and the occupancy of collagen fibers in the upper dermis (excluding the grenz zone) that were visualized by MT staining and found that they were significantly negatively correlated (*ρ* = −0.383; p = 0.0002; n = 90; Spearman’s rank correlation coefficient) (Figure 6). These findings suggest that increased epidermal MMP-1 and decreased dermal collagen fibers caused by photoaging occurred during the same period.

**Figure 7:**
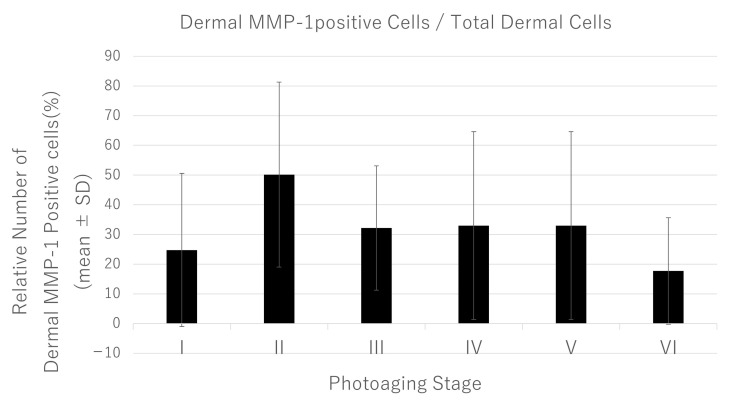
Changes in the number of dermal MMP-1-positive cells during vary with the progression of photoaging-related dermal tissue remodeling. The graph shows the average number of dermal MMP-1-positive cells in the total number of DAPI-stained nuclei in dermal cells (%), excluding hair follicle cells at each photoaging stage (stages I to IV). Graph data represent the mean ± SD of three fields of five samples of each photoaging stage (total of ninety fields). A significant difference was not observed when comparing this with photoaging stage I (Tukey’s test). MMP-1, matrix metalloproteinase-1; SD, standard deviation.

Dermal MMP-1-positive cells were abundant at sites with reduced collagen fiber densities in skin at an early photoaging stage. The epidermal MMP-1 signal intensity in each epidermal area increased with the decrease in dermal collagen fibers of skin tissue that had been subjected to chronic sun exposure. However, no significant changes in the ratio of MMP-1-positive dermal cells to the total number of dermal cells with photoaging stage progression were observed (Figure 7). This finding suggested that the increased number of dermal MMP-1-positive cells was not attributed to sun exposure; rather, it is considered to result from collagen degeneration due to other factors.

**Figure 8:**
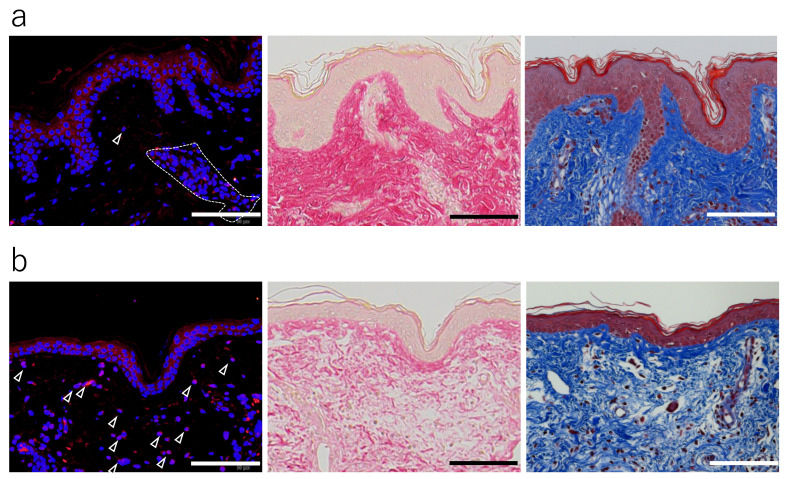
Differences in MMP-1-positive cells on skin tissues in the early photoaging stage between high and low collagen fiber densities. The correlation between the number of dermal MMP-1-positive cells and the collagen fiber density in tissue sections of photoaged skin. Images showing a high number of MMP-1-positive dermal cells and a low density of collagen fibers in skin stained with Elastica van Gieson and Masson’s trichrome (**a**), as well as a low number of MMP-1-positive dermal cells and a high density of collagen fibers (**b**). Clustered cells were excluded, because they were considered to be hair follicles (dotted areas). MMP-1 was visualized by immunofluorescence using an anti-MMP-1 antibody (red; arrowheads). Cell nuclei were counterstained with DAPI (blue). Bars: 100 μm.

**Figure 9:**
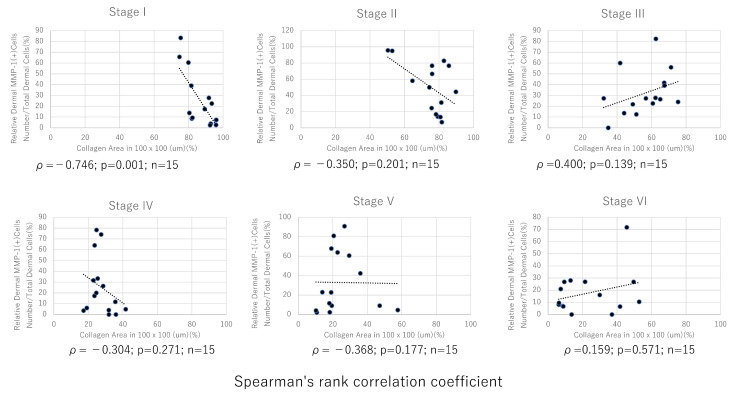
The correlation between the number of dermal MMP-1-positive cells and dermal collagen fiber density. Scatter plot showing the relationship between the number of dermal MMP-1-positive cells in the total number of dermal cells (%) and mean collagen occupancy rate (%) in the reticular dermis (data from Figure 3 are shown) at each photoaging stage (stages I to IV). Dotted line represents the regression line. Correlations between two values were analyzed by Spearman’s rank correlation coefficient. Significant negative correlations between two values were only detected in samples classified as photoaging stage I. MMP-1, matrix metalloproteinase-1.

A detailed analysis of the skin tissue sections revealed that areas of reduced collagen fiber density, visualized on sections that were stained with EVG or MT, exhibited a high number of MMP-1-positive cells in the dermis, as visualized by the anti-MMP-1 on adjacent sections (Figure 8). Therefore, we analyzed the relationship between the ratio of dermal MMP-1-positive cells to the total number of dermal cells and the occupancy of collagen fibers in the upper dermis (excluding the grenz zone) and detected a negative correlation between them, specifically in photoaging stage I (*ρ* = −0.746; p = 0.001; n = 15) (Figure 9). These results suggest that the presence of dermal-cell-derived MMP-1 is associated with a decrease in dermal collagen fibers, at least in skin classified as photoaging stage I.

## 4. Discussion

In this study, dermal collagen reduction caused by chronic sun exposure and its relationship with changes in MMP-1 expression in the epidermis were investigated. A histological analysis of 30 periauricular skin tissues revealed that skin classified as photoaging stages III and VI (moderate to highly advanced dermal tissue remodeling due to chronic sun exposure) had increased epidermal MMP-1 signal intensity in each epidermal area. Additionally, a negative correlation was detected between the epidermal MMP-1 signal intensity and the occupancy of collagen fibers in the upper dermis (excluding the grenz zone).

A generally recognized hypothesis regarding the mechanism of dermal tissue remodeling caused by chronic sun exposure is that repeated exposure and inadequate repair of skin damage due to solar UV exposure lead to the accumulation of damage and tissue remodeling, resulting in signs of photoaged skin [5,11]. Experiments that included human skin that had been irradiated with UV-B have shown that this damage is caused by MMPs originating from the epidermis. Although these MMPs can degrade collagen in the skin, the tissue remodeling observed in photoaged skin is mainly concentrated in the dermis. Keratinocyte-derived MMPs cross the epidermal basement membrane and diffuse into the dermis [5,9]. This study is the first to report the involvement of epidermal MMP-1 in the reduction in collagen fibers associated with dermal tissue remodeling caused by chronic sun exposure, which is consistent with previous reports on artificial UV-irradiated experiments [5,9].

MMP-1 also induces neutrophil chemotaxis through the release of interleukin-8 by human vascular smooth muscle cells [12]. The main product of activated neutrophils is neutrophil elastase [13]. Neutrophil elastase, a type of serine protease produced by neutrophils, is a potent proteolytic enzyme with the ability to degrade collagen and elastic fibers [14]. Increased MMP-1 in epidermis classified as photoaging stages III and VI may indirectly induce degradation of elastic fibers by chronically promoting neutrophil chemotaxis through its interaction with vascular smooth muscle in the dermis. Interestingly, the site at which the elastotic material first arises [4] and the site of vessels with smooth muscle layers are both deep in the dermis.

Studies that included cultured cells and ex vivo skin showed that UV-A stimulation, as well as UV-B stimulation, accelerates MMP-1 expression in dermal fibroblasts [15,16,17]. However, in skin subjected to chronic sun exposure, the infiltration of MMP-1-positive T lymphocytes in the dermis correlates with the severity of collagen degeneration, as estimated by reviewing images of EVG-stained skin tissue sections [18]. Additionally, changes in the ratio of M1 macrophages to M2 macrophages are involved in the loss of collagen in photoaged skin [19]. Therefore, the effects of dermal-cell-derived MMP-1 on dermal tissue remodeling induced by chronic sun exposure cannot be ignored. Our histological analysis revealed MMP-1 expression in the dermis. However, the ratio of dermal MMP-1-positive cells to the total number of dermal cells did not change with the progression of the photoaging stage.

This study had some limitations. Cell types were not distinguished in this study. However, if fibroblasts, T cells, and macrophages had been identified, certain characteristics might have been detected. Additionally, immunofluorescence staining using anti-MMP-1 showed the presence of MMP-1 in the extracellular matrix, and an analysis of the relationship between extracellular MMP-1 and dermal tissue remodeling could have provided more accurate findings. However, because of the difficulties associated with separating nonspecific signals, this relationship was not investigated.

Interestingly, at the same photoaging stage, a large standard deviation (i.e., interindividual differences) of the ratio of dermal MMP-1-positive cells to the total number of dermal cells was observed (Figure 7). This suggests that the percentage of dermal MMP-1-positive cells can vary even at the same photoaging stage of the skin. A negative correlation was detected between the percentage of dermal MMP-1-positive cells and the occupancy of collagen fibers in the upper dermis of skin classified as photoaging stage I. For example, the degradation of extracellular matrix components by increased MMP is critical for normal biological processes (e.g., wound healing and angiogenesis with inflammatory responses) [20]. These findings suggest that the presence of MMP-1-positive cells in the dermis is related to an immediate self-response that requires skin changes rather than tissue remodeling caused by chronic sun exposure.

The signaling mechanisms that resulted in excessive MMPs in the skin are not fully understood. However, it is believed that UV light and reactive oxygen species (ROS) generated by it directly or indirectly enhances the activity of mitogen-activated protein kinase family signaling cascades and activates transcription factor activator protein-1 to promote the production of MMPs [11,21,22,23]. Another transcription factor, nuclear factor-kappa B, is also activated by them and regulates the transcriptional activity of inflammation-related factors such as MMPs and cytokines [24,25,26]. Sun-exposure-induced DNA methylation enhances MMP-1 expression in human skin [27]. Additionally, signaling induced by matrix-bound cellular communication network factor-1, which is increased by UV irradiation, upregulates MMP-1 in dermal fibroblasts [28], suggesting that solar exposure promotes MMP-1 production through multiple mechanisms. Therefore, controlling the mechanisms by which sun exposure activates activator protein-1 and nuclear factor-kappa B, as well as UV-induced DNA methylation and cellular communication network factor-1-induced signaling in epidermal cells that promote MMP-1 production, could help protect against dermal tissue remodeling involving complex biochemical reaction cascades induced by chronic sun exposure.

The inhibitory effects of collagen peptides [29] and plant extracts [30] on MMP-1 activity have been observed during experiments using fibroblasts obtained from sun-exposed skin and UV-exposed cells. Prostaglandin inhibitors effectively suppress MMP-1 expression and enhance type 1 procollagen expression in UV-irradiated skin [31], suggesting that a mechanism that increases MMP-1 via COX2 may exist. Therefore, the application of these MMP-1-controlling agents to epidermal cells could suppress or improve chronic solar-exposure-induced dermal tissue remodeling. Oral administration of collagen hydrolysate for 4 weeks has been shown to improve the elasticity of sun-exposed skin [32].

In addition, it has been suggested that vitamin D exerts an antioxidant effect; in particular, the blood vitamin D level in patients with photo-induced skin cancer is significantly lower than that in non-cancer patients [33]. MMP-1 creates a microenvironment within tissues that are involved in the growth and invasion of cancer cells [34,35,36], and the overexpression of MMP-1 is also involved in carcinogenesis [37]. For example, the overexpression of MMP-1 that is specific to murine epidermis has been reported to be more sensitive to chemical carcinogens [37]. Thus, interindividual variation in antioxidant abilities in the epidermis may also cause interindividual variation in the expression of MMP-1 triggered by chronic sun exposure, resulting in interindividual variation in skin cancer risks. In addition to skin cancer, MMP-1 may be involved in the pathogenesis of other diseases; however, there are currently no confirmed reports on this matter.

With the extension of the average life span, skin aging is increasingly recognized not only as a cosmetic issue but also as a factor that compromises the mechanical protective function of the skin. Against this background, Dermatoporosis (DP) has been proposed as a concept that comprehensively includes the failure of skin function due to aging [38]. Risk factors of DP are aging and chronic sunlight [39]. Increased expression of MMPs and downregulation of tissue inhibitor of MMP-1 with aging lead to reduce dermal collagens and elastin [40], which have been implicated in DP [39]. The present findings suggest that increased epidermal MMP-1 due to chronic sun exposure contributes to the development of DP, as well as photoaging. However, to explain the direct relationship between epidermal and/or dermal MMP-1 and the reduction in dermal collagen fibers, validation experiments including animal models, such as MMP-13 knockout mice [41], are necessary. Such experiments should include models that mimic chronic sun exposure. Additionally, the contribution of MMP-1 in the dermal extracellular matrix, especially in elastotic materials, to collagen degeneration and dermal tissue remodeling caused by chronic sun exposure remains controversial, because it is difficult to distinguish between specific and nonspecific signals in elastotic materials using immunofluorescence. Moreover, photoaging stages III and IV in this study could not be clearly distinguished, because immunohistochemical stains using anti-decorin antibody were omitted. Therefore, data from detailed immunohistochemical analyses using specific antibodies that recognize collagen and various dermal cells are necessary to obtain precise results. Since only photoaged subjects are included in this study, comparisons with non-exposed subjects or those with minimal sun exposure may yield additional insights.

## 5. Conclusions

This study revealed that chronic exposure to sunlight results in increased MMP-1 in the epidermis. Additionally, epidermal MMP-1 induced by chronic exposure may be more involved than dermal MMP-1 in the reduction in collagen fibers associated with photoaging. However, the presence of dermal MMP-1-positive cells may be related to biological processes that require a short-term response during the early photoaging stage. When developing drugs to control the expression of MMP-1 in the skin, it is necessary to carefully consider the symptoms that are targeted for treatment and examine the effects on a variety of skin cells.

## Figures and Tables

**Table 1:** Subjects and skin tissue samples.

Participant	Sample	Photoaging Stage	Sampling Region	Sex	Age (Years)
1	1	I	Postauricular	Male	31
2	2-1	I	Postauricular	Female	76
3	3-1	I	Postauricular	Female	77
4	4-1	I	Postauricular	male	64
5	5-1	I	Postauricular	Male	39
6	6-1	II	Postauricular	Female	61
7	7-1	II	Postauricular	Male	39
8	8	II	Postauricular	Male	54
9	9-1	II	Postauricular	Female	64
10	10-1	II	Postauricular	Male	68
11	5-2	III	Preauricular	Male	39
12	6-2	III	Preauricular	Female	61
13	11-1	III	Preauricular	Female	70
14	11-2	III	Preauricular	Female	70
15	12	III	Preauricular	Female	77
16	2-2	IV	Preauricular	Female	76
17	3-2	IV	Preauricular	Female	77
18	9-2	IV	Preauricular	Female	64
19	13	IV	Preauricular	Female	83
20	14	IV	Preauricular	Female	83
21	4-2	V	Preauricular	Male	64
22	7-2	V	Preauricular	Male	69
23	15	V	Preauricular	Female	90
24	16	V	Preauricular	Male	67
25	17	V	Preauricular	Male	72
26	10-2	VI	Preauricular	Male	68
27	10-3	VI	Preauricular	Male	68
28	18	VI	Preauricular	Male	67
29	19	VI	Preauricular	Male	77
30	20	VI	Preauricular	Male	77

## Data Availability

The raw data used for statistical analysis in this study are available from the corresponding author upon reasonable request.

## Abbreviations

The following abbreviations are used in this manuscript:

EVG	Elastica van Gieson
MMP	matrix metalloproteinase
MT	Masson’s trichrome
UV	ultraviolet
